# Intelligent Tutoring Systems: Re-Envisioning Surgical Education in Response to COVID-19

**DOI:** 10.1017/cjn.2020.202

**Published:** 2020-09-10

**Authors:** Nykan Mirchi, Nicole Ledwos, Rolando F. Del Maestro

**Affiliations:** Neurosurgical Simulation and Artificial Intelligence Learning Centre, Montreal Neurological Institute and Hospital, McGill University, Montreal, Quebec, Canada; Faculty of Medicine, University of Toronto, Toronto, Ontario, Canada

**Keywords:** surgical education, surgical simulation, virtual reality, residency training, neurosurgery

COVID-19 has presented a series of unique challenges to surgical education. In the face of the global pandemic, access to traditional training techniques is limited. As health and safety have become top priorities, surgical residents have been reassigned to COVID units and most elective surgeries have been either postponed or cancelled. For surgical trainees, these reductions mean the loss of crucial learning opportunities to practice technical psychomotor skills. For residents in their ultimate or pen-ultimate years of residency who are soon to complete their final exams, this is a significant concern. While online resources for operative anatomy are available and training in emergency surgical cases continue, the reduction in elective cases presents an important problem for residents. Surgical educators are now faced with the challenge of ensuring their residents receive adequate training during this time. For both residents and surgical educators, it is impossible to predict how long this pandemic will last and what its effects on surgical training will be.

Advances in high-fidelity simulations provide residents with new opportunities to practice surgical skills and mitigate the challenges faced by current training paradigms including minimal access to standardized and objective feedback. These high-fidelity simulations were first extensively employed in the aviation industry, where trainees are required to practice and be examined on simulators before becoming accredited. However, the development and integration of virtual reality simulation in surgical education has been limited, especially in fields involving complex bimanual operative skills such as neurosurgery. This has resulted in a slow adoption of high-fidelity simulation technology paradigms for the assessment, training, and evaluation of trainees in some surgical fields.

The current global pandemic provides an opportunity to re-examine surgical education.^1^ As many technical skills training programs are temporarily put on hold, residents as well as medical students are concerned about their ability to acquire the necessary skillsets to allow them to provide excellent care to their patients. Simulators can be used to help trainees become familiar with specific surgical procedures, analyze 3D anatomical structures, and practice technical skills in risk-free environments. Intelligent tutoring systems are automated teaching platforms capable of providing individualized performance feedback to learners using a variety of simulators. These systems, in combination with virtual reality simulators, allow important surgical training to continue even during a pandemic.

Intelligent tutoring systems integrated with virtual reality simulation allow operators to experience hyper-realistic tool interactions with the anatomical tissue. These systems can harvest the power of large datasets and utilize machine learning to automatically differentiate surgical expertise and provide feedback on operative performance.^2–4^ The Virtual Operative Assistant, an intelligent tutoring system powered by machine learning for neurosurgical simulation, discusses the limitations and challenges of this technology.^5^ By deconstructing psychomotor skills into teachable metrics, trainees can obtain personalized feedback on specific factors identified by algorithms which may improve performance.

**Figure 1:**
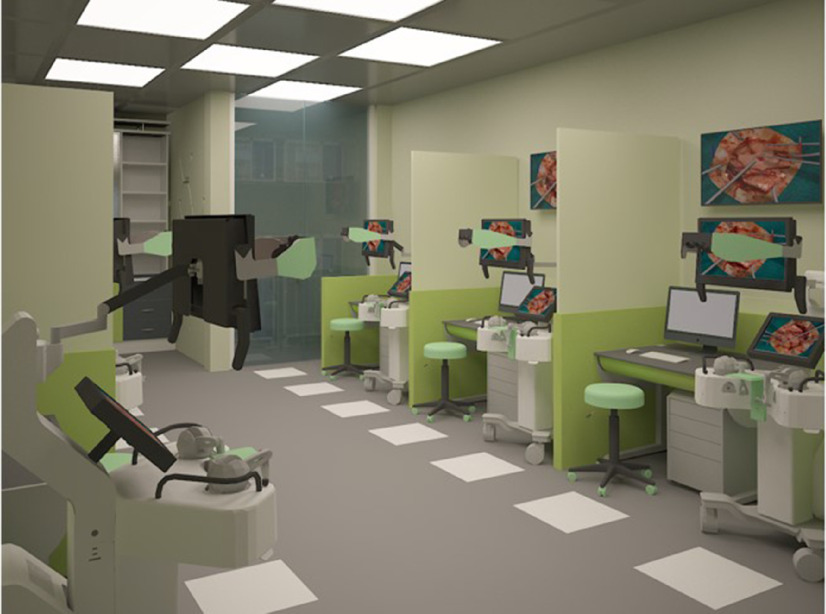
Prototype for a surgical simulation training center incorporating intelligent training systems. Trainees can practice a simulated procedure on the virtual reality platforms and obtain individualized feedback displayed on the monitor above each station. Simulators are arranged with sufficient space to maintain social distancing while limiting distraction. The figure was produced by Dr. Mostafa Sabbagh and Manal Al-Tahawi who have granted the co-authors permission of use.

An important factor when re-envisioning surgical education with automated teaching is how to accomplish a smooth integration of intelligent tutoring systems into current technical skills training. Dedicated learning centers could be incorporated within active hospital environments, allowing timely and easy access to learners interested in improving surgical psychomotor performance. Trainees could practice technical skills on the simulator to the level of mastery and obtain continuous feedback on performance in an iterative manner, with decreasing reliance on educator supervision. This learning-focused environment would provide trainees with an immersive and engaging way to practice and enhance their technical skills, all while practicing social distancing. A prototype for such a center is illustrated in Figure 1. We recognize that the cost associated with such a training center would not be insignificant. However, the value of their development and implementation need to be considered relative to the current toll of technical and procedural errors on patients and society.^6^ Mistakes in high-risk neurosurgical procedures also have high rates of medico-legal claims further increasing their impact on society.^7–9^ The potential that training in these specialized centers will aid in the understanding and prevention of surgical errors needs to be rigorously explored.

The authors are not advocating for the replacement of the present educational paradigms by automated systems. We recognize that human interaction is vital to learning. Rather, we believe that the integration and the wider availability of intelligent tutoring systems may complement present curricula while minimizing the impact of events such as a pandemic on trainees’ skills development. Surgical education programs will have to adapt in order to smoothly integrate automated and in-person education pedagogy. Intelligent tutoring systems can utilize a variety of simulation platforms to provide almost unlimited opportunities for repetitive practice without constraints imposed by the availability of supervision. In these risk-free environments, numerous adaptable and clinically relevant simulations can be tailored to the needs of learners, consistent with best practices for simulation-based education.^10–12^ These systems also increase the surgical educators’ armamentarium to help learners achieve mastery levels of surgical performance. Several studies are currently underway to compare the effectiveness of intelligent tutoring systems with more traditional formative teaching methods employed by surgical educators. The democratization of data accumulated from these simulation platforms is also important.^13^ As simulators become more available across international training centers, it would be important to establish a centralized database of anonymized simulation data. This database should be accessible by research groups across the globe to develop more representative and generalizable models to power intelligent tutoring systems. As we navigate through these unprecedented times, the full impact of COVID-19 on surgical education programs is yet to be ascertained. With continued research, increased development, and dissemination of intelligent tutoring systems, we can be better prepared for ever-evolving future challenges.
